# The Observable Movement Quality scale for patients with low back pain (OMQ-LBP): validity and reliability in a primary care setting of physical therapy

**DOI:** 10.1186/s12891-023-06784-1

**Published:** 2023-09-04

**Authors:** M. J. H. van Dijk, A. M. van der Wal, J. Mollema†, B. Visser, H. Kiers, Y. Heerkens, M. W. G. Nijhuis – van der Sanden

**Affiliations:** 1 Utrecht University of Applied Sciences, Institute of Human Movement Studies, Utrecht, the Netherlands; 2 Radboud University Medical CenterRadboud Institute for Health Sciences, IQ Healthcare, Nijmegen, the Netherlands; 3 Centre of Expertise Urban Vitality, Faculty of Health, Amsterdam University of Applied Sciences, Amsterdam, the Netherlands; 4 Department Occupation & Health, HAN University of Applied Sciences, Nijmegen, the Netherlands

**Keywords:** Movement quality, Low back pain, Activities, Standardized observational assessment, Physiotherapy, Exercise therapy

## Abstract

**Background:**

The Observable Movement Quality scale for patients with low back pain (OMQ-LBP) is a newly developed measurement instrument for use in primary care settings of physical and exercise therapists to assess movement quality (MQ) of patients with low back pain (LBP).

**Objective:**

This study aims to determine validity, reliability and feasibility of the OMQ-LBP. The OMQ-LBP consists of a standardized movement circuit (performed twice) consisting of five daily activities problematic for LBP patients, which are scored with an 11-item observation list.

**Methods:**

Construct validity was determined by testing seven hypotheses on associations between constructs (*n = *85 patients with LBP) and four hypotheses on known group differences (*n = *85 patients with LBP and *n = *63 healthy controls; *n = *35 matched participant-patients having VAS-pain ≥ 20 mm during and/or after both circuits and healthy controls). Internal consistency was analyzed with Cronbach’s alpha (*n = *85 patients with LBP). For inter- and intra-rater reliability Intraclass Correlation Coefficient (ICC) values were examined (*n = *14 therapists: seven primary care physical therapists and seven exercise therapists). Additionally, content validity and feasibility were determined using thematic analysis of a brief interview with participants, patients (*n = *38) and therapists (*n = *14).

**Results:**

After Bonferroni correction 2/7 associations between constructs and 2/4 significant group differences were confirmed. Cronbach’s alpha was 0,79. The ICC-values of interrater reliability of the OMQ-LBP total score and the duration score were 0.56 and 0.99 and intra-rater reliability 0.82 and 0,93, respectively. Thematic analysis revealed five themes. Three themes elucidate that both patients and therapists perceived the content of the OMQ-LBP as valid. The fourth theme exhibits that OMQ-LBP provides a clear and unambiguous language for MQ in patients with LBP. Theme 5 depicts that the OMQ-LBP seems feasible, but video recording is time-consuming.

**Conclusions:**

The OMQ-LBP is a promising standardized observational assessment of MQ during the five most problematic daily activities in patients with LBP. It is expected that uniform and objective description and evaluation of MQ add value to clinical reasoning and facilitate uniform communication with patients and colleagues.

**Supplementary Information:**

The online version contains supplementary material available at 10.1186/s12891-023-06784-1.

## Introduction

Low back pain (LBP) is the leading cause of disability in West European countries [1] and the most treated health problem in Dutch primary care settings of physical therapists and exercise therapists [2]. Pain and problems in performing or maintaining routine activities, such as standing, climbing, lifting, or walking, restrict the participation in daily life, work, and leisure of patients with LBP [3–7]. Observation and analysis of movement quality (MQ) – the way a person moves – are key elements in the design, choice and evaluation of interventions [8–11]. The Dutch "Guideline low back pain and lumbosacral radicular syndrome" mentions the observation of MQ as a key point throughout diagnostic and therapeutic reasoning [12].

Skjaerven's Movement Quality Model (MQM) emphasizes that the quality of how a person moves represents a synthesis of biomechanical, physiological, psycho-socio-cultural, and existential processes [13]. This multidimensionality is recognized in clinical practice where MQ in patients with LBP is linked to all domains of the International Classification of Functioning, Disability and Health (ICF) [14, 15]. While performing daily activities, e.g. picking up an object, most patients with LBP show a consistent adapted lumbar movement pattern. It is suggested that such a reduction in variability of movement strategies is related to activity limitations and is seen as a risk factor for chronic LBP [5, 16, 17].

In clinical practice activities of patients with LBP are often assessed with Patient Reported Outcome Measures (PROMs) [12, 18], such as, the Patient Specific Complaints questionnaire (PSC) and the Quebec Back pain Disability Scale (QBPDS) [7, 12, 19–23]. These questionnaires establish an overview of the perceived limitations in activities and restrictions in participation from a patient’s perspective. Currently, a standardized observational assessment of MQ during relevant problematic activities is lacking [10, 20, 24]. This hampers comparison of the therapeutic observations with the patient’s experience and the physical examination of for instance the mobility of the spine. Moreover, physical and exercise therapists differ in their observation, description, and interpretation of MQ in patients with LBP [8, 10].

The Observable Movement Quality scale for patients with Low Back Pain (OMQ-LBP) for therapists’ aims to achieve a standardized observation and an uniform and objectified description and evaluation of how the patient performs activities. The development of the OMQ-LBP is based on two extended inventories in clinical practice [10, 14] and a systematic review [24].

The OMQ-LBP consists of a movement circuit and a standardized observation list. In the movement circuit, the patient performs five daily activities. These activities have shown discriminative value in MQ between patients with LBP and healthy controls [24, 25], and are mentioned by patients with chronic LBP as most difficult in daily living [20]. The standardised observation list of 11 items describes and evaluates MQ. Additionally, the duration of the circuit performance is assessed.

The OMQ-LBP assessment complements the PROMs, like the Patient Specific Complaints questionnaire (PSC) or Quebec Back Pain Disability Scale (QBPDS) and the physical diagnostic examination [22, 23]. Therefore, we expect that the OMQ-LBP supports the biopsychosocial approach of physical and exercise therapists [12].

This study aims to determine the validity, reliability and feasibility of the OMQ-LBP for use in primary care settings of physical and exercise therapists.

## Methods

**Fig. 1:**
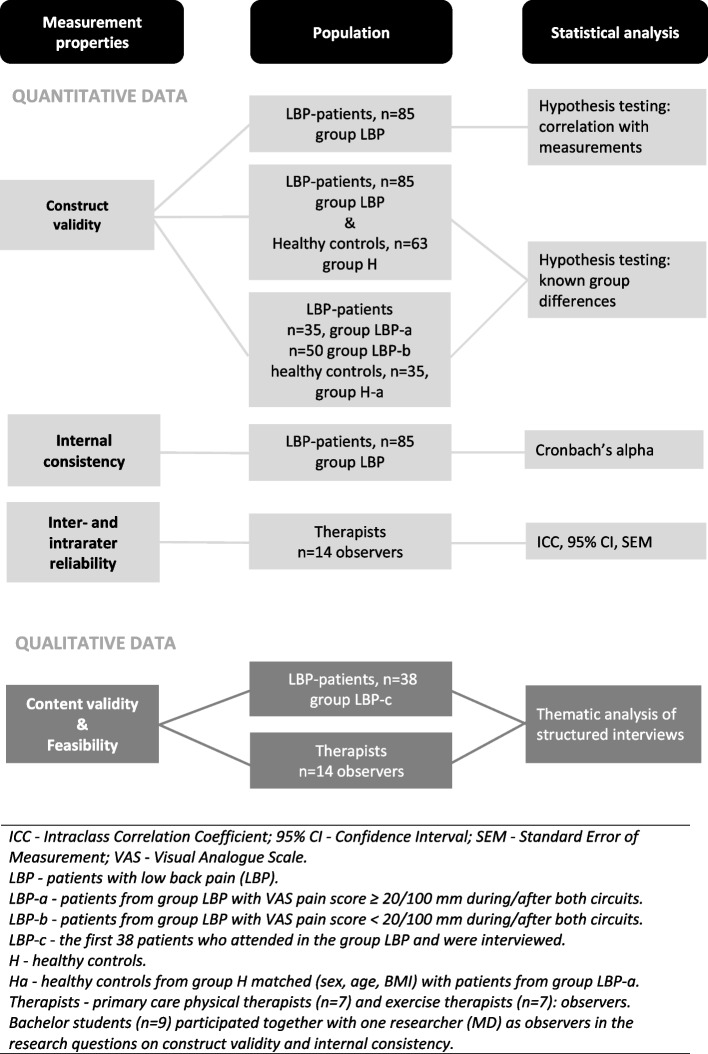
Flow diagram

Construct validity was tested with a priori-formulated hypotheses and internal consistency was determined with Cronbach's alpha. For testing inter- and intra-rater reliability the Intraclass Correlation Coefficient (ICC) with 95% Confidence Interval (95% CI) and the Standard Error of Measurement (SEM) were determined. Furthermore, content validity was explored through a thematic analysis of interviews with participant-patients and therapists. These interviews also gave insight into the feasibility of the OMQ-LBP. See Fig. 1.

### Scoring the OMQ-LBP scale

The OMQ-LBP scale includes 11 items to assess observable qualitative aspects of movements. These movements are video-recorded as participants walk along an 8-shaped circuit (4 m) and consecutively perform the activities: 1) getting up from a chair; 2) picking up a coin from the floor, turning around a stool and putting the coin back on the floor; 3) lifting a crate that contains five 1 L plastic bottles filled with water, carrying the crate, and putting it down on the stool; 4) walk to the chair and sit down, then again get up from the chair, walk towards the mat behind the stool and lie down for three seconds on the back with extended legs; and 5) get up and sit down on the chair again. Participants were instructed to move in their preferred way and at their own pace. Appendix 1 provides details on camera positioning, distance, and instructions. The videos were recorded in MP4 or MOV files.

The 11 items are scored separately for both the first and second circuit on a five-point Likert-scale. Each score represents the frequency with which a certain qualitative aspect was observed. Per circuit the scores range between 11–55. The OMQ-LBP total score ranges from 22–110. A higher total test score indicates a higher MQ. The duration of each circuit is measured with a stopwatch. The total duration score is the number of seconds of both circuits.

### Participants

#### Patients with LBP

From March 2020 until October 2021 a convenient sample of patients, age > 18 years with non-specific and specific LBP was invited to participate by primary care physical and exercise therapists, and by bachelor students who attended their internship in these practices. LBP defined as pain located between the lower ribs margins and the buttock creases and is commonly accompanied by pain in one or both legs or by associated neurological symptoms in the lower extremities [26]. Persons with any central neurological or major circulatory or respiratory disorder, pregnancy, hip or knee arthritis, or with a Body Mass Index (BMI) ≥ 30, were excluded.

#### Healthy controls

Recruitment of healthy controls was initially planned during a public information day at the HU-UAS in March 2020. However, due to COVID-19 government measures, this approach was cancelled shortly before the event. Subsequently, a convenient sample of healthy controls was recruited from the researcher (MD) and the bachelor students’ professional and social network. This lasted until September 2021. Inclusion criteria for healthy controls were age > 18 years old and not having had LBP in the past two years. Exclusion criteria were any central neurological or major circulatory or respiratory disorder, pregnancy, hip or knee arthritis, or a BMI ≥ 30.

Upon signing the consent form, the participant-patients and healthy controls provided demographic information (gender, age, BMI, non-specific of specific LBP, duration of LBP and experienced pain (mean and highest of the last week). The movement circuit was then explained and participants had a practice run to ensure that they understood the instructions. Subsequently, they performed the circuit twice to obtain a total test score. Patients’ assessments occurred in nine private practices in Culemborg, Kampen, Krimpen aan de Lek, Tilburg, Twello, Utrecht, Waalre, Wilp, and Zoetermeer, and in the movement science lab at the HU-UAS. Healthy controls were tested in six private practices in Culemborg, Eindhoven, Kampen, Waalre, Zoetermeer, and Zwolle, and in the movement science lab at the HU-UAS.

#### Observers

Students and therapists participated as observers.

Nine students (eight male, mean age 24,8 (± 1,9)) who were completing their bachelor's education in physical therapy or exercise therapy at the HU-UAS participated as observers. The students signed for confidentiality and provided their gender and age. The videos were recorded by all students (10 participants per student) and one researcher (MD). The video-recordings were observed and scored by five students (14 participants per student) and one researcher (MD). The students had no prior experience with the OMQ-LBP and were not familiar with the participants to be observed. However, they were not blinded to participants’ status (patient or healthy control).

From March to May 2021 primary care physical and exercise therapists employed in Dutch primary care settings, were verbally informed about the interrater and intra-rater reliability study and the total time investment of approximately eight hours over ten weeks. Those who were interested in participating as observers and treated at least five patients with LBP per month in the past year received an invitation letter. The therapists had no prior experience with the OMQ-LBP and were blind to the subjects’ status. After signing confidentially and providing their gender, age and work experience the therapists were invited for the training and were given access to a secure research folder in the Utrecht University of Applied Science research drive (HU-RD).

An independent data-steward (JM) facilitated and guarded the data sampling.

#### Training OMQ-LBP

In an extra 2-h session the students were trained to: inform the participants about the study’s purpose, obtain informed consent, record demographics, explain and video-record the movement circuit, administer questionnaires, securely store data in the HU-RD and delete the recordings from their mobile phones.

The students and therapists received a 4-h training that aimed: 1) explaining the purpose of the OMQ-LBP; 2) understanding the items and item definitions and scoring the items by watching video-recordings; 3) measuring circuit duration with mobile phone stopwatch; 4) explaining privacy rules with regard to watching video-recordings of the participants; and 5) collecting data securely in HU-RD. During the training, observers watched and scored at least five video-recordings of patients with LBP and one healthy subject. Individually scores were compared and discussed, addressing any understanding of the definitions and any differences in the scoring were used to calibrate scoring among observers. The video-recordings, OMQ scale-scoring file and training manual were available in personal HU-RD folders. So, the observers could get familiar with the HU-RD. One researcher (MD) trained the students. The training of the physical (*n = *7) and exercise therapists (*n = *7) was provided by a master student (RH) and a researcher (MD). The master student had four years of work experience as a physical therapist and was pursuing a master's degree in manual therapy at the HU-UAS.

### Measurement instruments of associated constructs

#### Visual analogue scale (VAS)

In LBP research this general scale is commonly applied to measure self-reported pain intensity on a 100 mm long horizontal line [27, 28]. Scores vary between ‘no pain at all’ (score 0) and ‘most imaginable pain’ (score 100). The VAS was applied to measure pain intensity during and after the movement circuit (VAS-P).

Additionally, a separate VAS (VAS-T) was used to assess the perceived potential damage to the lower back during performance of the circuit. A score of 0 represents ‘no hazard of damage’ and the score of 100 indicates ‘very high damage potential’.

#### Tampa scale for kinesiophobia (TSK-13)

The TSK assesses patient's fear of injury and its influence on avoiding movements [29]. We choose the TSK-13 version to reduce participants burden. The TSK-13 is strongly associated with lifting performance [30] and covers the subscales harm factor and avoidance activity, relevant for this study. We expect that beliefs about serious bodily harm negatively influences movement quality and may lead to activity avoidance [31]. Each item has four answer options ranging from ‘strongly disagree’ to ‘strongly agree’. The total sum score ranges from 13–52, with a higher score indicating a greater fear of movement [29, 30].

#### Borg rating of perceived exertion scale (Borg-RPE)

The Borg-RPE scale measures perceived exertion during physical stress. Scores range from 6 ‘no effort’ to 20 ‘absolute maximum efforts’ [32, 33]. Although psychometric properties of the Borg-scale are unknown it is mentioned as suitable for use in patients with chronic non-specific LBP [34].

#### Patient specific complaints questionnaire (PSC)

The PSC is a questionnaire used to report personal complaints as result of back problems during activities [12, 22]. Patients can choose three activities that are difficult or impossible to perform and rate the degree of difficulty on a 10 points scale ranging from 0 = 'able to perform the activity without problems' to 10 'unable to perform the activity'. The scores of the three activities are summed (range 0–30). A higher score indicate more difficulties with performing activities [22, 35].

#### Quebec back pain disability scale (QBPDS)

The QBPDS is a valid and reliable questionnaire used to measures functional status in patients with LBP [12, 23, 36–38]. It consists of six sub-domains that assess functional skills namely, bed rest, sit-stand, walking, moving, bending over, and moving heavy objects. Each item is scored on a 6-point likert scale. The total sum score ranges from 0 (no limits) to 100 (completely limited).

### Construct validity

No gold standard is available to test the same construct of MQ in patients with LBP. In the absence of a gold standard, the COSMIN guideline advises to test hypotheses on correlations between outcomes of instruments measuring related but dissimilar constructs and known group differences [39–41]. These correlations are expected to be low [40]. We tested seven hypotheses on associations between MQ and related but dissimilar constructs. Below and in Table 2 we explain the direction and magnitude of the expected correlations. Moreover, we examined four hypotheses on differences between patients with LBP and healthy controls.

#### A. Associations between constructs

LBP reduces velocity of movement [24, 42]. Therefore, lower OMQ-LBP total scores were expected to associate with longer duration of the movement circuit (Table2, hypothesis 1). Although many individual variations, pain and pain-related fear in patients with LBP are considered to limit adaptability in motor control e.g., less flexion in the lumbar spine during lifting [16, 43, 44]. This might negatively influence MQ during the activities of the circuit. Therefore, lower OMQ-LBP total scores were anticipated to associate with higher levels of experienced pain and pain-related fear (Table 2, hypotheses 2 and 3), assessed using VAS-P [28] and TSK-13 [29],respectively. Task‐specific fear, one’s thoughts that the performance of the activities of the movement circuit might damage their lower back, also might negatively influence MQ during the circuit [45]. Therefore, lower OMQ-LBP total scores were expected to associate with experiencing these thoughts (Table 2, hypothesis 4), assessed using VAS-T. Exercise tolerance, a body function related to respiratory and cardiovascular capacity as required for enduring physical exertion [15], is relevant in LBP management [3, 46]. For analysing MQ, physical and exercise therapists take notice of nonverbal expression of exertion e.g., the effort required to perform the activity [10]. Therefore, lower OMQ-LBP total score were expected to associate with a higher level of experienced exercise tolerance (Table 2, hypothesis 5), assessed using the Borg-RPE [32]. Because the constructs tested in hypotheses 1–5 are dissimilar from MQ and participant-patients will differ with respect to velocity of movement, levels of pain, pain-related fear, and perceived exertion, weak to moderate correlation (0.2–0.4) were expected [47, 48] (Table 2, hypotheses 1–5).

Patients with LBP have difficulty performing and/or sustaining everyday activities such as standing, lifting and walking [5, 20, 49, 50]. Therefore, lower OMQ-LBP total scores were expected to associate with higher levels of perceived low back complaints. The level of functioning was assessed with the PSC [22] and the QBPDS [23, 37]. Due to observed activities of the circuit might only partial overlap the activities of the PSC and items of the QBPDS and divers levels of functioning among participant-patients, weak to moderate correlations (0.2–0.4) were expected [47, 48] (Table 2, hypotheses 6–7).

#### B. Known group differences

Compared to healthy controls, patients with LBP have different motor control strategies and proprioception and slower movement [24, 42]. Therefore, we hypothesized that compared to healthy controls patients with LBP have significantly lower OMQ-LBP scores and longer duration scores for the movement circuit (Table 3, hypotheses 8 and 9). Movement velocity is seen as an aspect of MQ in patients with LBP [10]. Therefore, participant-patients with VAS-P score of < 20 mm during and after both circuits were expected to have discomfort rather than pain [51], while participant-patients having VAS-P scores of ≥ 20 mm would have more pain leading to greater differences in the OMQ-LBP total score and total duration scores compared to those patients with VAS-P scores < 20 mm and compared to healthy controls [10, 42]. Therefore, patients having VAS-P ≥ 20 mm during and/or after both movement circuits (*n = *35) were matched (gender, age, BMI) with 35 healthy controls (Table 3, hypotheses 10–11).

To determine construct validity, 75% of the hypotheses should be confirmed [39, 47].

### Internal consistency

To determine the internal consistency of the OMQ- LBP the degree of interrelatedness of its items with the total score was determined using data from all participant-patients (*n = *85, group LBP in Fig. 1 and Table 1).

**Table 1:** Demographics of the participant-patients and healthy controls

Demographics	Participant-patients and healthy controls						
	**LBP** *n = *85	**LBP-a** *n = *35	**LBP-b** *n = *50	**LBP-c** *n = *38	**H** *n = *63	**H-a** *n = *35	**V** *n = *12
**Male/female**^**^**^	37/48 44/56%	14/21 40/60^#^%	24/26 48/52%	14/24 37/63%	29/34 46/54%	14/21 40/60%	6/6 50/50%
Age in years^*^	42,6 (± 16,6)	42,8 (± 17,4)	42,4 (± 16,2)	42,5 (± 16,4)	39,0 (± 21,2)	42,4 (± 17,0)	46,0 (± 14,8)
BMI^*^	23,6 (± 2,9)	23,2 (± 3,0)	23,8 (± 2,8)	22,7 (± 2,4)	23,6 (± 2,2)	22,5 (± 2,6)	23,5 (± 2,2)
**Non-specific LBP/specific LBP**^**^**^	66/19 78/22%	29/6 83/17%	40/10 80/20%	29/9 76/24%	-	-	6/4^#^ 60/40%
Duration LBP in month^*^	114,3 (± 132,7)	106,8 (± 152,1)	119,6 (± 118,6)	110,0 (± 137,3)	-	-	226,0^#^ (± 84,8)
VAS-pain: mean last week^*^	27,6 (± 17,6)	35,9 (± 18,5)	21,9 (± 14,5)	25,7 (± 16,4)	-	-	24,7^#^ (± 16,2)
VAS-pain: highest last week^*^	48,2 (± 24,6)	61,6 (± 20,3)	38,9 (± 23,2)	44,7 (± 23,5)	-	-	47,0^#^ (± 36,0)

LBP - patients with LBP

LBP-a—patients from group LBP with a VAS-pain score ≥ 20/100 mm during and/or after two circuits

LBP-b—patients from group LBP with VAS pain score < 20/100 mm during and/or after both circuits

LBP-c—the first 38 patients who attended in the group LBP and were interviewed

H—healthy controls

H-a—healthy controls from group H, matched (sex, age, BMI) with patients from group LBP-a

V Video-recordings of selected participants of group LBP (*n = *10^#^) and of group H (*n = *2)

*LBP* low back pain, *BMI* Body Mass Index, *VAS* Visual Analogue Scale (0–100 mm)LBP—patients with LBP

^^^Frequency and percentage

^*^Mean (standard deviation)

Each item of the OMQ-LBP scale represents a different element of MQ. Together, these items contribute to the construct of MQ in patients with LBP, which is considered a formative measurement model. In a formative measurement model, individual items may not necessarily correlate with each other, unlike a reflective measurement model where items are manifestations of the construct. So, in a reflective measurement model item correlation is expected or items can be interchangeable [39, 52]. In a formative measurement model items are not interchangeable, however, it is expected that they correlate with the total score. At this development stage, identifying all items that contribute to the construct is most important [39].

### Inter- and intra-rater reliability

To determine inter- and intra-rater reliability 12 video-recordings of 10 participant-patients and two healthy controls (group V in Table 1) were purposively selected by a researcher (MD) from a sample of the first 61 video-recorded participant-patients and 29 healthy controls. To represent variety in clinical practice, selection of video-recordings were based on gender, age and OMQ-LBP scores.

**Fig. 2:**
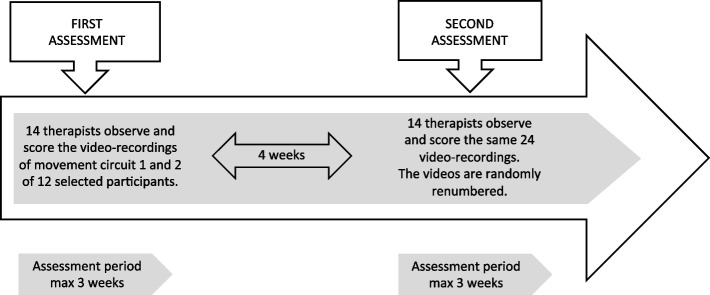
Procedures form the intra-and interrater reliability study Interrater reliability = first assessment; intra-rater reliability = first & second assessment

Each therapist received a personal number and a link to a folder in the HU-RD. This folder contained video-recordings of both circuits of 12 participants, along with corresponding numbered scoring sheets. The automatic counting of the scoring of OMQ-LBP scale scores and duration ensured accuracy of data recording. The assessment procedure of the therapists is described in Fig. 2.

To ensure timely completing of all video-recordings two researchers (RH and MD) guided the process via e-mail and organized the materials for each therapist in the HU-RD.

To calculate the ICC of the interrater reliability the scores of therapists’ initial assessment were used. To determine intra-rater reliability the scores of the first and second assessment were applies. See Fig. 2.

### Content validity and feasibility

In the primary developmental stage of the OMQ-LBP, content validity was based on two questionnaire studies and a literature review [10, 14, 24]. To determine if the OMQ-LBP also reflects patients’ experiences and therapists expertise, structured interviews were taken [39, 53, 54]. The initial 38 participating-patients (group LBPb in Fig. 1 and Table 1), were interviewed by five students (*n = *19) and one researcher (MD) (*n = *19) after completing the movement circuit and filling out the questionnaires. After finishing their assessment procedure, therapists (*n = *14) were interviewed by one researcher (MD) at the therapist's practice.

The structured interview focused on content of the OMQ-LBP and its feasibility in primary care settings of physical and exercise therapists consisting of seven open questions [55, 56]. See Appendix 3. The taped interviews were saved in the HU-RD and transcribed by one researcher (MD).

### Statistical analysis

To determine internal consistency, a sample of at least 110 patients was sought (10 participant-patients per item). To get a good impression of reliable use of the OMQ-LBP in clinical practice, we assumed that 14 therapists would represent the diversity of therapists in clinical practice. Based on this assumption, we calculated the sample size for participants performing the circuit with fixed alpha and power values on 0.05 and over 80, respectively. In addition, we set the null correlation and alternative correlation at the ICC values 0.3 and 0.6, respectively [57, 58]. This obtained a sample size of 11 video-recordings of participants performing the circuit. To realize a varied composition regarding gender, age, MQ level and duration of the circuit as well as including two healthy subjects, we choose to select 12 video-recordings.

Descriptive statistics of the demographics of the participants, students, and therapists and scores of the OMQ-LBP scale, duration of the movement circuit, VAS-P during and after the movement circuit, TSK-13, VAS-T, Borg-RPE scale, PSC, and QBPDS were calculated. Categorical variables are presented as numbers and percentages, while continuous data is reported as means and standard deviations (SD).

In case of bivariate normal distribution, Pearson’s correlation coefficients were used to test hypotheses 1 to 7, while Spearman’s rho was considered when variables were not normal distributed. P-values and 95% CI were reported. Correlations were interpreted weak (< 0.3); weak to moderate (> 0.2 < 0.4); moderate (> 0.3 < 0.7); moderate to high (> 0.6 < 0.8); high (> 0.7) [47]. To analyze the group differences the Mann–Whitney test (hypotheses 8–9) and a one-way ANOVA and post hoc tests based on Bonferroni (hypotheses 10–11), were employed. A *p*-value of ≤ 0.05 was considered significant. To account for multiple testing the α level for hypotheses 1–9 (Tables 2 and 3) was adjusted using the Bonferroni-method [59, 60]. CI-95% and effect sizes were reported.

**Table 2:** Hypotheses on correlations between measurement outcomes

Hypotheses on correlations between measurement outcomes	Scores of patients with LBP (*n = *85)^**^**^	Correlations
1. Longer duration of the movement circuit (more seconds) will have weak to moderate negative correlation (-0.2 to -0.4) with lower OMQ-LBP total scores	OMQ-LBP scores: 87,9 (± 10,5) [57–109] Duration: 112,4 (± 18,2) [0, 3–170] seconds	Spearman: -0.47 *p* = 0.000^#^ 95% CI: -0.625 to -0.279
2. Higher pain scores during (a) and after (b) performing the movement circuits will have weak to moderate negative correlation (-0.2 to -0.4) with lower OMQ-LBP total scores	a. VAS-P during the circuit: 17,1 (± 20,2) [0–77] b. VAS-P after the circuit: 18,0 (± 20,7) [0–79]	a. During: Spearman -0.12 *p* = 0.268^#^ 95% CI: -0.332 to 0,100 b. After: Spearman -0.13, *p* 0.221 95% CI: -0.343 to 0.088
3. Higher TSK-13 scores will have weak to moderate negative correlation (-0.2 to -0.4) with lower OMQ-LBP total scores	TSK-13: 23,0 (± 7,6) [13–57]	Spearman -0.19 *p* = 0.069^#^ 95% CI: -0.400 to 0.022
4. Higher VAS-T scores will have weak to moderate negative correlation (-0.2 to -0.4) with lower OMQ-LBP total scores	VAS-T: 11,8 (± 19,8) [0–82]	Spearman -0.17§ *p* = 0.119^#^ 95% CI: -0.375 to 0.051
5. Higher scores for the perceived exertion during two movement circuits will have weak to moderate negative correlation (-0.2 to -0.4) with lower OMQ-LBP total scores	Borg-RPE: 8,5 (± 2,0) [6–13]	Pearson -0.33 *p* = 0.002^#^ 95% CI: -0.510 to -0.129
6. Higher PSC scores will have weak to moderate negative correlation (-0.2 to -0.4) with OMQ-LBP total scores	PSC: 14,7 (± 5,9) [1–26]	Pearson -0.22 *p* = 0.044^#^ 95% CI: -0.413 to -0.006
7. Higher QBPDS scores will have weak to moderate negative correlation (-0.2 to -0.4) with lower OMQ-LBP total scores	QBPDS: 23,0 (± 12,3) [1–56]	Pearson -0.25 *p* = 0.024^#^ 95% CI: -0.435 to -0.033

*OMQ-LBP* Observable Movement Quality scale in patients with Low Back Pain, *LBP* low back pain, *p* significance, *95% CI* 95% Confidence interval, *VAS-P* Visual Analogue Scale (0–100 mm) to assess the experienced pain during and after the movement circuit, *TSK-13* Tampa Scale for Kinesiophobia-13 items, *VAS-T* Visual Analogue Scale (0–100 mm) to assess participants’ thoughts that the performance of activities of the movement circuit might damage their lower back, *Borg-RPE* Borg Rating of Perceived Exertion, *PSC* Patient Specific Complaints questionnaire, *QBPDS* Quebec Back Pain Disability Scale

^^^Mean scores (standard deviation)[range]

^#^After Bonferroni-method the α level of 0.05 was adjusted to 0.005

**Table 3:** Hypotheses on known group differences

Hypotheses on known group differences	Scores of patients with LBP (*n = *85) and healthy controls (*n = *63)^**^**^	Differences
8. The total OMQ-LBP total scores of the patients with LBP will be significantly lower than the OMQ-LBP total scores of the healthy controls	OMQ-LBP total score patients: 87,9 (± 10,5) [57–109] OMQ-LBP total score healthy controls: 102,1 (± 4,7) [84–110]	Mann–Whitney U *p* = 0.000^#^ 95% CI: -16.0 to -12.0
9. The duration scores of the movement circuit of the patients with LBP will be significantly longer than the duration scores of the healthy controls	Duration patients: 112,4 (± 18,2) [0, 9–170] seconds Duration healthy controls: 105,4 (± 12,3) [0–140] seconds	Mann–Whitney U *p* = 0.026^#^ 95% CI: 0.64 to 9.59
Hypotheses on known group differences: subgroups	Scores of LBP-patients experiencing higher (A)° and lower pain (B) during/after the circuit and healthy controls (C)°	Differences^*^
10. The difference between the OMQ-LBP score of LBP-patients with VAS-P ≥ 20/100 mm during/after two circuits (A) and matched healthy controls (C) is expected to be larger than the difference between the OMQ-LBP score of LBP-patients with VASP < 20/100 mm during/after two circuits (B) and healthy controls (C)	OMQ-LBP total score: A: 85,6 (± 13,4) [57–109], *n = *35^ B: 89,3 (± 7,8) [75–107], *n = *50 C: 102,7 (± 4,5) [84–110], *n = *35^	A-C: *p* < .001 B-C: *p* < .001 A-B: *p* .261 No larger difference
11. The difference between the total duration score of LBP-patients with VAS-P ≥ 20/100 mm during/after two circuits (A) and matched healthy controls (C) is expected to be larger than the difference between the total duration score of LBP-patients with VAS-P < 20/100 mm during/after two circuits (B) and healthy controls (C)	Total duration score in seconds: A: 119,2 (± 21,1) [0, 3–170], *n = *35^ B: 107,6 (± 14,4) [9–162], *n = *50 C: 103,2 (± 10,2) [0, 9–110], *n = *35^	A-C: *p* < .001 B-C: *p* .606 A-B: *p* .003 Compared to the mean difference between B-C, the mean difference between A-C is 11,56 s larger

*OMQ-LBP* Observable Movement Quality scale in patients with Low Back Pain, *LBP* low back pain, p significance, *95% CI*-95% Confidence interval, *VAS-P* Visual Analogue Scale (0–100 mm) to assess the experienced pain during and after the movement circuit, *s* seconds

°Matched pairs (*n= *35)

^^^Mean scores (standard deviation)[range]

^#^After Bonferroni-method the α level of 0.05 was adjusted to 0.005

^*^Bonferroni was integrated into the statistical test

To establish internal consistency Cronbach’s alpha was analyzed and the item contribution to the total score was reported. Correlations ≥ 0.3 are considered to contribute to the total score. Cronbach’s alpha values between 0.70 and 0.90 are well accepted for internal consistency [39, 59].

For testing interrater reliability the ICC was rated with the two-way random-effects single-measures model of absolute agreements with 95% CI, along with the SEM [39, 48]. The intra-rater reliability ICC was rated with the two-way mixed model of absolute agreement with 95% CI, along with the SEM [14, 17]. The SEM was estimated with the formula: standard deviation of the mean difference x √2 [39]. ICC values less than 0.5 are indicative of poor reliability, values between 0.5 and 0.75 indicate moderate reliability, values between 0.75 and 0.90 indicate good reliability, and values greater than 0.90 indicate excellent reliability [59].

Statistical analyses were performed with the IBM Statistical Package for the Social Sciences (IBM SPSS Statistics), version 25 (IBM Corp, Armonk, NY, USA).

Thematic analysis was conducted independently by two researchers (AW and MD) [61] using ATLAS.ti 22. The analysis involved five steps: 1) reading the interviews and familiarizing with the data; 2) systematically generating initial codes; 3) organizing codes into themes, with close reference to both research questions content validity and feasibility; 4) cross checking themes and codes; and 5) identifying patterns within and across the data of the patients and therapists to refine and define theme similarities [61]. During and after each step the researchers explored similarities and discussed the differences. Disagreements were solved with a third researcher (YH).

## Results

### Participants

A total of 85 patients with LBP and 63 healthy controls participated. See the flow diagram (Fig. 1). The demographics are provided in Table 1 (participant-patients, healthy controls) and in the text (students and therapists).

### Construct validity

#### A. Associations between constructs

Findings supported the association between observed MQ on the one hand, and movement velocity and perceived physical exertion, on the other (Table 2, hypotheses 1 and 5). However, no associations were found with pain, fear, and patient’s thoughts of potential damage to the lower back (Table 2, hypotheses 2, 3 and 4). Two hypotheses were confirmed by weak non-significant correlations between observed MQ and experienced difficulties while performing activities, and MQ and self-reported functional status (Table 2, hypotheses 6–7).

#### B. Known group differences

Both hypotheses regarding lower OMQ-LBP total scores and longer duration scores in patients with LBP compared to healthy controls were confirmed (Table 3, hypotheses 8–9), indicating distinct movement quality and velocity. Compared to patients with VAS-P < 20/100 mm during/after two circuits patients experiencing more pain had a larger difference of duration scores relative to healthy controls. This difference was not observed in the OMQ-LBP total score (Table 3, hypotheses 10–11).

### Internal consistency

Cronbach’s alpha showed the observation list to reach moderate reliability α 0,79 (*n = *85 participant-patients, group LBP in Fig. 1 and Table 1). Except for item 3 *‘moving symmetrically’* and item 11 ‘*activities can be performed’* the item-total correlations were above α 0.30 (Table 4).

**Table 4:** Item contribution to the total OMQ-LBP score

OMQ-LBP Observation list	Item-Total Correlation
Item 1. Moving fluently	0,52
Item 2. Secondary movements	0,57
Item 3. Moving symmetrically	0,23
Item 4. Rotations while moving	0,49
Item 5. Moving stereotypically	0,58
Item 6. Range of motion of joints	0,58
Item 7. Use of muscle strength	0,47
Item 8. Muscle tone	0,60
Item 9. Respiration	0,41
Item 10. Pain behaviour	0,50
Item 11. Activities can be performed	0,17

*OMQ-LBP* Observable Movement Quality scale in patients with Low Back Pain, *LBP low back pain*

### Inter- and intra-rater reliability

Seven physical and seven exercise therapists (mean age 34,7 (± 12,0) years, nine male) with a mean of 11,8 (± 16,2) years of work experience participated. The therapists provide monthly an average of 31,0 (± 2,8) interventions to patients with LBP in a primary care setting across the Netherlands. The demographics of the twelve participants selected for the video-recordings are provided in Table 1 (group V).

All therapists completed the OMQ-LBP scores. One therapist forgot to measure the duration of the circuit for one patient and one therapist recorded the duration of the video-recordings instead of the exact duration of the circuit. Therefore, reliability analysis of the OMQ-LBP total score and duration score was based on the data of 14 and 12 therapists, respectively. Inter- and intra-rater reliability of the OMQ-LBP total score showed moderate and good ICC-values, respectively. For the duration scores of the circuit the ICC-values of the inter- and intra-rater reliability were excellent. See Table 5.

**Table 5:** Inter- and intra-rater reliability

	ICC	CI -95%	SEM
**Interrater reliability**^#^			
- OMQ-LBP total score^*^	0,56	0,35 - 0,79	3,8
- Duration of the movement circuit^^^	0,99	0,97 - 0,10	3,6
**Intra-rater reliability**			
- OMQ-LBP total score^*^	0,82	0,49 - 0,95	1,8
- Duration of the movement circuit^^^	0,93	0,80 - 0,98	3,4

*OMQ-*LBP Observable Movement Quality scale in patients with Low Back Pain, *ICC—*Intraclass Correlation Coefficient, *CI-95%* 95% confident intervals, *SEM* Standard Error of Measurement

^#^The calculation of the interrater reliability was based on the total test scores of the 1^st^ assessment (see Fig. 2)

^*^n = 14 observer-therapists

^**^**^*n = *12 observer-therapists

### Content validity

A total of 38 patients and 14 therapists were interviewed. The mean interview duration was 5,8 (1, 9) minutes for patients and 24,8 (3,3) minutes for therapists. During step 2 and 3, researchers (AW, MD) resolved their questions and disagreements. In steps 4 and 5 the third researcher (YH) provided support in checking the theme patterns and theme definitions. The themes were approved by all researchers.

**Fig. 3:**
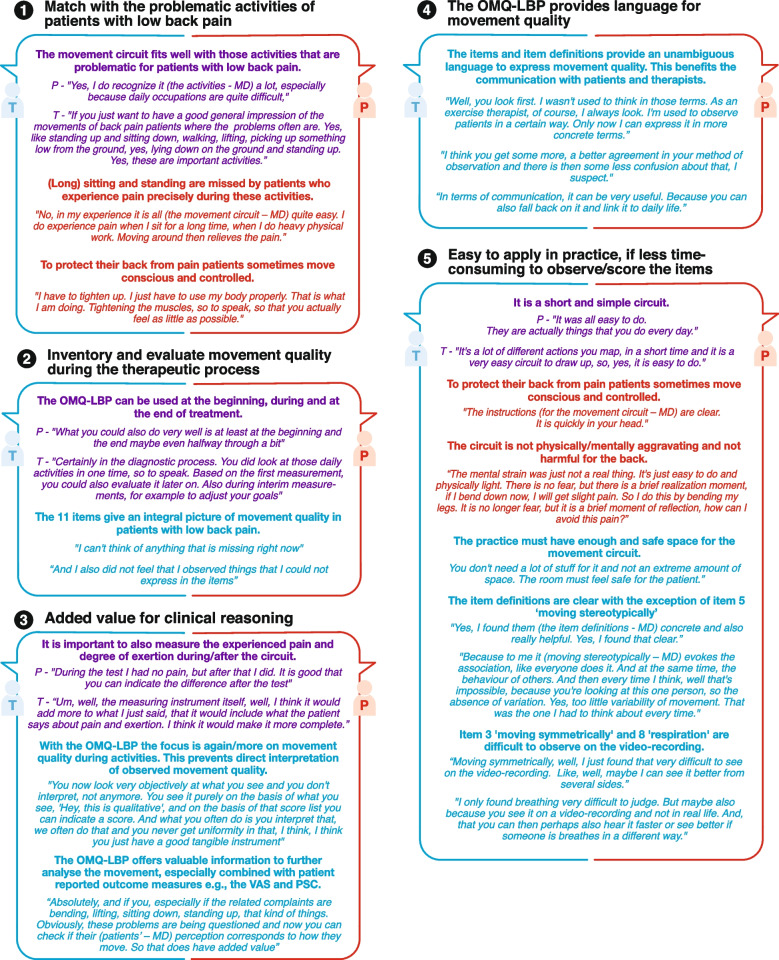
The five themes resulting from the thermatic analysis • Topic content. ○ Topic feasibility. P-Patients; T-Therapists. OMQ-LBP − Observable Movement Quality scale for patients with Low Back Pain: VAS—Visual Analogue Scale: PSC—Patient Specific Complaints: MD—researcher

Thematic analysis revealed five themes from the interviews (Fig. 3). The first theme shows patients’ and therapists’ agreement that the movement circuit activities correspond to problematic activities for patients with LBP. However, patients mentioned missing long lasting positions leading to pain e.g., standing and sitting. Moreover, patients reported that they move in a controlled way. The second and third themes indicate that both patients and therapists found that the standardized observation is helpful in understanding and explaining the relationship between MQ and patients' complaints, especially when questioning pain and exertion during and after the movement circuit. It also facilitates comparison with PROM’s, which supports the process of clinical reasoning. The fourth theme highlights for therapists that OMQ-LBP provide clear and unambiguous language to share ideas about MQ in patients with LBP and with colleagues. The fifth theme depict that OMQ-LBP is feasible for use in primary care settings of physical and exercise therapists. However, the video-based observation and scoring of MQ were time-consuming. The therapists recommended a course on explaining item definition, and judgeless observation and scoring of the items. Figure 3 illustrates the themes with quotes.

## Discussion

This study aimed to establish validity, reliability and feasibility of the newly developed OMQ-LBP. In line with the COSMIN criteria, we conclude that the OMQ-LBP is a promising assessment for clinical use [40]. Since, patients with LBP and primary care physical and exercise therapists have confirmed content validity, internal consistency is acceptable and intra-rater reliability for the OMQ-LBP total score was good and while inter- and intra-rater reliability for the duration scores was excellent. However, not all hypotheses were statistically significant confirmed. Meaning that construct validity needs further examination. Moreover, interrater reliability for the OMQ-LBP scores needs improvement. Patient and therapists expect the application of the OMQ-LBP to be feasible in clinical practice.

### Validity

The confirmed distinctive qualities regarding observed MQ and movement velocity between patients and healthy controls and between patients with < VAS-P 20/100 mm ≥ (hypotheses 8 and 11) and the correlations between OMQ-LBP total and the duration scores and OMQ-LBP and the Borg-RPE scores (hypotheses 1 and 5) are consistent with the principle that pain alters motor control and movement velocity [16, 24, 42, 62–64].

No correlations were found between OMQ-LBP and TSK-13 scores and between OMQ-LBP and VAS-T scores (hypotheses 2–4). Similarly, scores of the Standardized Mensendieck Test (SMT) and the Body Awareness Rating Scale Movement Quality and Experience (BARS-MQE) do not correlate with pain and pain-related anxiety [63, 65, 66]. The SMT and BARS-MQE could not be used as gold standard because both tests primarily assess MQ on functionality as judged by the therapist compared to a normative correct performance during static postures and walking [65, 66]. Contrary the OMQ-LBP does not focus on functionality but describes relevant characteristics of MQ e.g., fluency and symmetry relative to the task and environment in patients with low back pain.

It seems important to reflect together with the patient on (dis)congruencies between the therapists’ observation related to the way the patient moves and the experienced difficulties as described in the PROMS. It opens the door for a reflection on patients’ experiences, cognitions and emotions. The importance of such reflections with the patient are recognized in LBP management [12, 67]. It is important to note here that patients indicated that complaints with prolonged postures, for example standing and sitting, are not covered in the OMQ-LBP. This needs special attention in clinical practice to interpret the observed MQ compared to patients' answers to those PROMs that pay attention to these activities.

### Reliability

Established acceptable internal consistency of the OMQ-LBP reflects the item-total correlations of a formative measurement instrument. All items in this formative measurement instrument have their own identity within the MQ construct and contribute to assessing MQ [39]. As LBP progresses, it is conceivable that observable qualitative movement aspects change and that the 11 items score differently at the beginning, during and at the end of an episode. Moreover, unchanged MQ over time is also a relevant signal [68, 69]. Therefore, all items are valuable to capture MQ in patients with LBP.

While intra- and interrater reliability for measured duration scores were excellent intra-rater reliability for the OMQ-LBP total score was better than interrater reliability, which was also found in studies focusing on the Observable Movement Quality scale (OMQ) that measures movement quality in children with mild to moderate motor impairments [70]. The determined moderate interrater reliability for the OMQ-LBP total score is in line with reliability values of common active movement tests e.g., waiters bow [71]. Probably, variety in the background of the participating therapists, and related tacit knowledge and perception on MQ may have negatively affected OMQ-LBP’s inter-rater reliability [72–74].

### Limitations and strengths

Firstly, the participant-patients were briefly informed about the purpose of the OMQ-LBP and the study procedure. To help participant-patients answer the second interview question (see Appendix 3), the interviewers spontaneously explained the purpose of the observation list further. This additional information may have influenced participants' answers.

Secondly, analysis of internal consistency was based on a smaller than intended number of participant-patients. However, the results showed that this did not prevent us from drawing conclusions.

Thirdly, during the training the therapists caught themselves giving a clinical interpretation to the observation of how a patient moves, e.g., MQ is good or bad. This entanglement of observation and interpretation is recognized in the results of the questionnaire study [13, 15]. for instance they do not describe the lack of rotation in the back, but describe their observation as an interpretation like, pain-avoiding movement Such unintentional interpretations of observed MQ may still have influenced the scoring of the OMQ-LBP item and total scores. The entanglement of observation and interpretation may also have influenced the students’ scoring of the OMQ-LBP. After all, they were not blind to the LBP status of the participants. Unraveling observation and interpretation requires updating the manual and training to increase interrater reliability.

After Bonferroni correction, not all predicted correlations between outcome measures and known group differences were significant. However, according to COSMIN [41], correct prediction of direction and magnitude is more important than significant results. This does not alter the fact that for use in clinical practice, additional validation research such as examining associations with motor control tests, other blind observers, and subgroups of people with LBP is needed. Moreover, responsiveness and clinical relevance should be investigated in a longitudinal study.

Strengths of this study include the comprehensive spectrum of examined measurement properties, alignment with COSMIN and adequate sample sizes. Moreover, there is a substantive fit with practice. Employing expertise from patients and both physical and exercise therapists and examining PROMs also used in specific LBP guideline ensures good generalizability [12].

### Clinical implication

For use in clinical settings, we recommend performing two circuits per assessment and that therapists score the 11 items immediately after both circuits. It is conceivable that item 3 ‘*moving symmetrically’* and item 9 ‘*breathing*’ are more observable through direct observation of MQ during the circuit. This contributes to a reliable observation of MQ. Moreover, the definition of item 5 ‘*moving stereotypically’* requires clarification. However, these practical adjustments require a review of measurement properties.

Given the nature of the OMQ-LBP, it is important to realize that the OMQ-LBP score provides a judgment-free description of MQ in patients with LBP. In the process of clinical reasoning, the observation of MQ complements PROMs and physical diagnostic tests. Uniform MQ descriptions help clarify the complexity of the relationship between observation and clinical reasoning [75]. This supports reconsidering and examining hypotheses to explore how observed MQ should be interpreted and might contribute to setting, realizing and evaluating patients' goals [76–78]. Both participant-patients and therapists indicated that questioning patients' perceived pain and physical exertion during and after both circuits provides additional insight into patients' movement strategies. To complete this picture, problematic activities that are absent from both circuits [20] and environmental factors [79] can be discussed as well. Because the item definitions clearly express MQ, they could also initiate a discussion about patients' experiences, beliefs and pain-related anxiety regarding the performance of activities. Sharing these experiences and beliefs might help individual patients make sense of pain [12, 49, 67, 80]. It might also support specific patient education and encourage self-management in patients with LBP [81]. Such clear and personalized LBP management meets patients’ expectations [82]. Therefore, we believe that the OMQ-LBP contributes to the assessment of physical functioning in patients with LBP and fits therapists' biopsychosocial approach [12, 18, 83, 84]. We recommend including the OMQ-LBP in the guideline on LBP for physical and exercise therapists and in therapist training [12]. Training in the use of defined observation criteria will improve the validity and reliability of measurements [52]. Such training is common for observational instruments.

### Scientific implication

Currently, the concept MQ is a niche in scientific research. We believe that an objective formulated outcome resulting from observation of MQ during activities that are problematic for patients with LBP could also add value to LBP research.

## Conclusion

The OMQ-LBP is a promising standardized observational assessment of MQ during the five most problematic daily activities in patients with LBP. The objectified description of MQ fits well in the therapeutic approach of primary care physical and exercise therapists, adds value to clinical reasoning, and facilitates uniform communication with patients and colleagues. Following this exploratory development and validation of the OMQ-LBP, further validation and examination of responsiveness is recommended.

### Supplementary Information

**Additional file 1:** **Appendix 1.** OMQ-LBP movement circuit.

**Additional file 2:** **Appendix 2.** OMQ-LBP observation list.

**Additional file 3:** **Appendix 3.** Interview participant-patients and therapist kopie.

## Data Availability

The datasets generated and analysed during the current study are not publicly available because we did not ask participants for permission to publish those data, but are available from the corresponding author upon reasonable request.

## Abbreviations

OMQ-LBP: Observable Movement Quality scale in patients with Low back Pain
MQ: Movement Quality
LBP: Low Back Pain
BMI: Body Mass Index
COSMIN: COnsensus-based Standards for the selection of health Measurement INstruments)
MQM: Movement Quality Model
HU-UAS: Utrecht-University of Applied Sciences
HU-RD: Research drive of the Utrecht-University of Applied Science
HC: Healthy control
VAS-P: Visual Analogue Scale for pain
VAS-T: Visual Analogue Scale for thoughts
TSK-13: Tampa Scale for Kinesiophobia
Borg-RPE: Borg Rating of Perceived Exertion scale
PSC: Patient Specific Complaints questionnaire
QBPDS: Quebec Back Pain Disability Scale
ICC: Intraclass Correlation Coefficient
95% CI: 95% Confidence Interval
SEM: Standard Error of Measurement
SMT: Standardized Mensendieck Test
BARS-MQE: Body Awareness Rating Scale Movement Quality and Experience
OMQ: Observable Movement Quality scale (generic movement quality in children)
