# Supplementary material for: The Observable Movement Quality scale for patients with low back pain (OMQ-LBP): validity and reliability in a primary care setting of physical therapy
Source: BMC Musculoskelet Disord. 2023 Sep 4;24:705. doi: 10.1186/s12891-023-06784-1 (PMC10476334; doi:10.1186/s12891-023-06784-1)
Supplement: Supplementary file 1 — Additional file 1: Appendix 1. OMQ-LBP movement circuit. [file 12891_2023_6784_MOESM1_ESM.pdf]

Appendix 1

OMQ-LBP movement circuit: a schematic presentation and the instructions

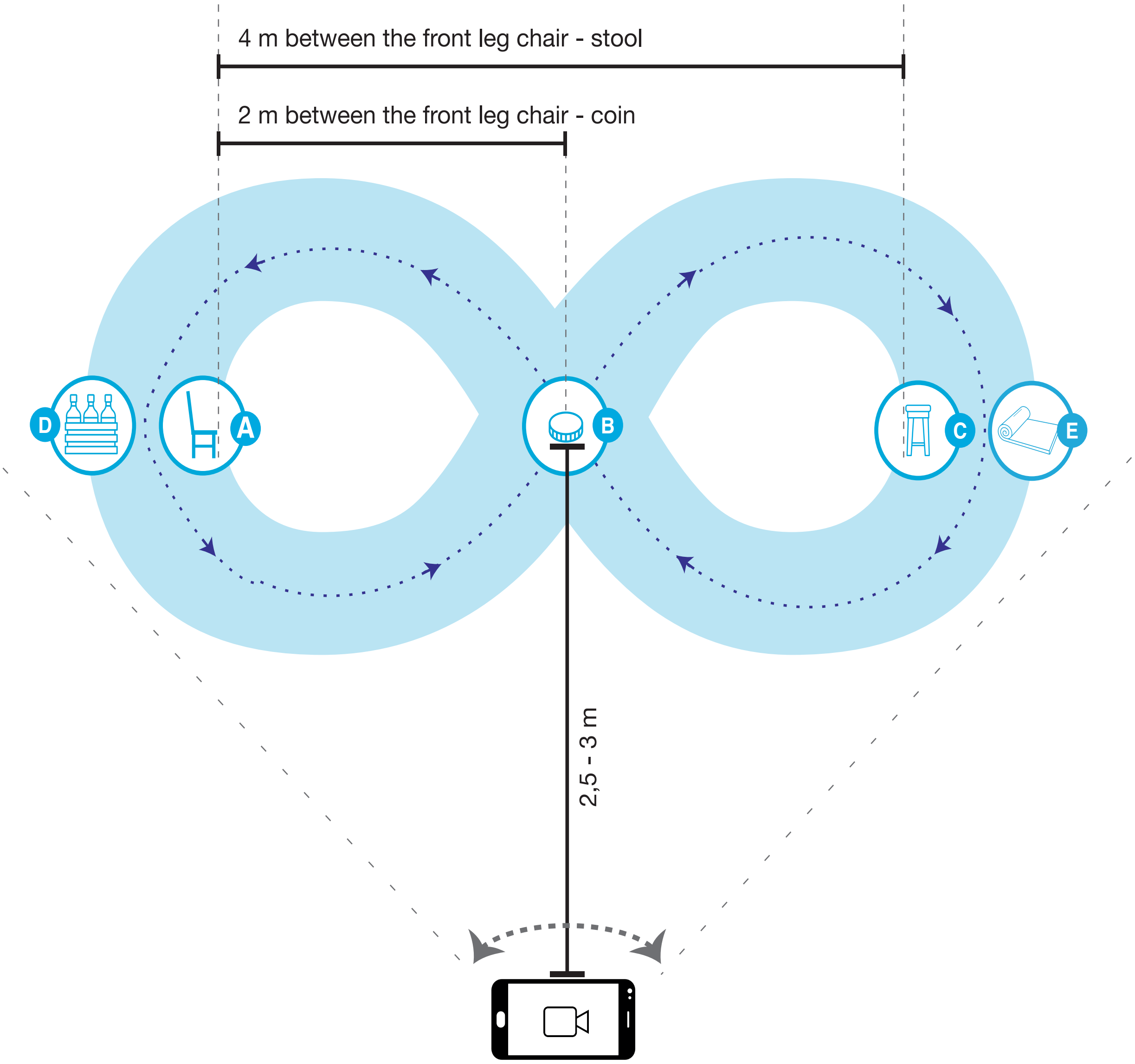

Camera height

Adjust the camera height per participant so that he/she is completely in the picture..

INSTRUCTION

The participants were instructed to move on their own way (as accustomed at home) and at their own preferred/comfortable pace. The circuit is performed and scored twice.

The circuit starts at A sitting on the chair; get up from the chair and walk to B. Pick up the coin-from the floor at B and continue on your way and walk behind the stool at C back towards B, put the coin back on the floor at B and follow the 8-shape further towards the crate at D. Lift the crate, that contains 5x 1l plastic bottles with water, standing behind the chair and put it on the stool at C and walk back to A and go to sit on the chair. Get up from the chair again, walk to E and go to lie on the mat and remain lying on your back with both legs straight for three counts. Stand up and go to sit on the chair again at A. The circuit is now finished.

LEGENDA

OMQ-LBP - Observable Movement Quality scale for patients with Low Back Pain
