# Supplementary material for: The Observable Movement Quality scale for patients with low back pain (OMQ-LBP): validity and reliability in a primary care setting of physical therapy
Source: BMC Musculoskelet Disord. 2023 Sep 4;24:705. doi: 10.1186/s12891-023-06784-1 (PMC10476334; doi:10.1186/s12891-023-06784-1)
Supplement: Supplementary file 2 — Additional file 2: Appendix 2. OMQ-LBP observation list. [file 12891_2023_6784_MOESM2_ESM.pdf]

Appendix 2

Observable Movement Quality scale for patients with Low Back Pain: observation list*.

1. Moving fluently

The person performs the movements of the various activities in such a way that they proceed without hesitations or interruptions or without acceleration/deceleration in terms of tempo/ rhythm.

1. Consistently not fluent
2. Typically not fluent; fluent in 1-3 phases of the activities
3. Half of the time not fluent; half of the time fluent
4. Mostly fluent; not fluent in 1-3 phases of the activities
5. Consistently fluent

2. Secondary movements

The person performs the various activities without any secondary movements, such as taking support with the hands on the legs or taking an extra step.

1. Consistently secondary movements
2. Typically secondary movements; no secondary movements in 1-3 phases of the activities
3. Half of the time secondary movements; half of the time no secondary movements
4. Mostly no secondary movements; secondary movements in 1-3 phases of the activities
5. Consistently no secondary movements

3. Moving symmetrically

The person moves in such a way that the direction of movements is aligned within the central axis during various activities, and without any lateral side bending of the trunk (spine, chest, pelvis).

1. Consistently not symmetrical
2. Typically not symmetrical; symmetrical in 1-3 phases of the activities
3. Half of the time not symmetrical; half of the time symmetrical
4. Mostly symmetrical; not symmetrical in 1-3 phases of the activities
5. Consistently symmetrical

4. Rotations while moving

The person performs the movements in such a way that trunk rotations expected for the activities occur.

1. Consistently no expected rotations
2. Typically no expected rotations; expected rotations in 1-3 phases of the activities
3. Half of the time no expected rotations; half of the time expected rotations
4. Mostly expected rotations; no expected rotations in 1-3 phases of the activities
5. Consistent expected rotations

5. Moving stereotypically

The person performs the movements of the various activities in a consistent manner.

1. Consistently stereotypical
2. Mostly stereotypical; not stereotypical in 1-3 phases of the activities
3. Half of the time stereotypical; half of the time not stereotypical
4. Typically not stereotypical; stereotypical in 1-3 phases of the activities
5. Consistently not stereotypical

6. Range of motion of joints

The person moves in such a way that the range of motion of joints matches with the required activities and with environmental factors relevant for these activities.

1. Consistently no appropriate match of the range of motion of joints
2. Typically no appropriate match of the range of motion of joints; appropriate match of the range of motion of joints in 1-3 phases of the activities
3. Half of the time appropriate match of the range of motion of joints; half of the time no appropriate match of the range of motion of joints

4. Mostly appropriate match of the range of motion of joints; no appropriate match of the range of motion of joints in 1-3 phases of the activities
5. Consistently appropriate match of the range of motion of joints

7. Use of muscle strength

The person moves in such a way that the use of muscle strength is appropriate for the required activities and matches the environment.
1. The use of muscle strength is consistently not appropriate
2. The use of muscle strength is typically not appropriate; in 1-3 phases of the activities the use of the muscle strength is appropriate
3. The use of muscle strength is not appropriate half of the time; half of the time the use of the muscular strength is appropriate
4. The use of muscle strength is mostly appropriate; in 1-3 phases of the activities the use of the muscle strength is not appropriate
5. The use of muscle strength is consistently appropriate

8. Muscle tone

The person moves in such a way that the muscle tone is appropriate for the required activities and the environment. Increased or decreased muscle tone is considered not appropriate.
1. Consistently no appropriate muscle tone
2. Typically no appropriate muscle tone; muscle tone appropriate in 1-3 phases of the activities
3. Half of the time no appropriate muscle tone; half of the time appropriate muscle tone
4. Mostly appropriate muscle tone; no appropriate muscle tone in 1-3 phases of the activities
5. Consistently appropriate muscle tone

9. Respiration

The person breathes regularly. The location and frequency of respiration matches the activities.
1. Consistently irregular respiration/location and frequency of respiration do not match the activities.
2. Typically irregular respiration/location and frequency of respiration do not match the activities; regular respiration/location and frequency of respiration matches the activities in 1-3 phases of the activities
3. Half of the time irregular respiration/location and frequency of respiration do not match the activities; half of the time regular respiration/location and frequency of respiration matches the activities
4. Mostly regular respiration/location and frequency of respiration matches the activities; irregular respiration/location and frequency of respiration do not match the activities in 1-3 phases of the activities
5. Consistently regular respiration/location and frequency of respiration matches the activities.

10. Pain behaviour

The person performs the activities without specific pain behaviour, such as, consciously (exaggerated) controlled moving, bracing, rubbing, grimacing and/or sighing.
1. Consistently pain behaviour
2. Typically pain behaviour; no pain behaviour in 1-3 phases of the activities
3. Half of the time pain behaviour; half of the time no pain behaviour
4. Mostly no pain behaviour; pain behaviour in 1-3 phases of the activities
5. Consistently no pain behaviour

11. The activities can be performed

The person can perform the required movements of the activities.
1. Consistently not fully performed
2. Typically not fully performed; only fully performed during 1-3 phases phases of the the activities
3. Half of the time the activities are fully performed; half of the time the activities are not fully performed
4. Most activities are fully performed; 1-3 phases of the the activities are not fully performed
5. Consequently fully performed

*

To translate the Dutch Observable Movement Quality scale for patients with Low Back Pain observation list used in this study into English, forward and backward translation was used (1, 2). Firstly, two native speakers performed an independent forward translation. One translator had no knowledge of the concept (DC) and the other translator was an expert in LBP management (JL). Together with a researcher (MD), a consensus version of both translations was developed. Secondly, two professional translators (MF, CvB) independently performed a backward translation to Dutch. Thirdly, an expert committee, consisting of a physical therapist (RR) and an exercise therapist (NS) compared the backward translation with the original version, and in consultation with three researchers (BV, MD, YH), the English translation of the observation list was completed.

References

1. Beaton DE, Bombardier C, Guillemin F, Bosi Ferraz M. Guidelines for the Process of Cross-Cultural Adaptation of Self-Report Measures. Spine (Phila Pa 1976). 2000;25(24):3186–3191.
2. Mokkink LB, Terwee CB, Patrick DL, Alonso J, Stratford PW, Knol DL, et al. The COSMIN checklist for assessing the methodological quality of studies on measurement properties of health status measurement instruments: An international Delphi study. Qual Life Res. 2010;19(4):539–49.
