# Supplementary material for: The Observable Movement Quality scale for patients with low back pain (OMQ-LBP): validity and reliability in a primary care setting of physical therapy
Source: BMC Musculoskelet Disord. 2023 Sep 4;24:705. doi: 10.1186/s12891-023-06784-1 (PMC10476334; doi:10.1186/s12891-023-06784-1)
Supplement: Supplementary file 3 — Additional file 3: Appendix 3. Interview participant-patients and therapist kopie. [file 12891_2023_6784_MOESM3_ESM.pdf]

Appendix 3

Interview particpant-patient and interview therapist.

Questions for the structured interview with the participant-patient.

CONTENT

1. Relevance of activities.
What is your opinion on the activities of the movement circuit? Do these activities match the daily activities that are problematic for you during the day, night, or week?
If not: which of your daily activities would you describe as problematic? Do you have suggestions for activities to add to the movement circuit?

2. Measuring movement quality.
Do you think that using this movement circuit could help your therapist for your treatment?

3. Completeness.
Do you consider the measurement instrument complete?
What is your view on completeness regarding the movement circuit, the observation list, and the scoring method?

FEASIBILITY

4. Clarity of instructions.
What is your opinion on the instruction of the circuit?
If the participant thinks the instructions are either clear or unclear, they can be asked to give a short explanation or illustrate this with an example.

5. Mental load of the movement circuit.
Are you able to correctly perform the activities of the circuit?
Do you experience pain during one or more activities of the circuit?
If pain occurred: during which activities did you experience the most/least pain? Are there one or multiple activities that were easy or difficult to perform due to the severity of pain?

6. Physical load of the movement circuit.
Would you consider the completion of the circuit easy or light? More specifically: would you consider it physically exhausting?

7. Test duration of movement circuit.
What is your opinion on the duration for completing the movement circuit?

Questions for the structured interview with the therapist

● CONTENT

1. Relevance of activities.
What is your opinion on the activities of the movement circuit?

2. Clarity.
What is your opinion on the observation items, the item definitions, and the scoring method?

3. Completeness.
Do you consider the measurement instrument complete?
What is your view on completeness regarding the movement circuit, the observation list, and the scoring method?
Do you have suggestions to add / delete / change something to the measurement instrument?

4. Therapeutic process.
What is your opinion on the idea to observe and score the movement quality in a standardized way, using the 11 items?
What does the use of this measurement instrument mean for clinical reasoning: in the diagnostic phase, in the therapeutic phase, and for the evaluation?

○ FEASIBILITY

5. Test duration of movement circuit.
What is your opinion on the duration for completing the movement circuit?
How much time did it take to assess the score for movement quality using the observation list?
Concerning the test duration: do you consider the measurement instrument to be practicable for your therapeutic practice?

6. Applicability of the measurement instrument.
What do you think about the applicability in practice of the measurement instrument for quality of movement?
How do you consider this applicability regarding: the movement circuit, the observation list, the scoring method, and the patient group?

7. Communication.
What would the measurement instrument mean to you regarding the communication with: patients and colleagues?
